# Intracellular Trafficking of the Human Cytomegalovirus-Encoded 7-*trans*-Membrane Protein Homologs pUS27 and pUL78 during Viral Infection: A Comparative Analysis

**DOI:** 10.3390/v6020661

**Published:** 2014-02-10

**Authors:** Ina Niemann, Anna Reichel, Thomas Stamminger

**Affiliations:** Institute for Clinical and Molecular Virology, University of Erlangen-Nuremberg, Schlossgarten 4, Erlangen 91054, Germany; E-Mails: ina.niemann@viro.med.uni-erlangen.de (I.N.); anna.reichel@viro.med.uni-erlangen.de (A.R.)

**Keywords:** HCMV, cytomegalovirus, 7-transmembrane protein, G protein-coupled receptor, GPCR, US27, UL78, virus infection, localization

## Abstract

Human cytomegalovirus (HCMV) encodes four G protein-coupled receptor (GPCR) homologs, termed pUS27, pUS28, pUL33, and pUL78. In contrast to the extensively characterized vGPCRs pUS28 and pUL33, knowledge concerning pUS27 and pUL78 is limited. Previous studies already demonstrated constitutive internalization of pUS27 and pUL78, as well as an association with the endosomal machinery, however, these results were mainly obtained using transiently transfected cells. To explore the subcellular localization of both receptors during viral infection, we constructed recombinant HCMVs expressing tagged vGPCRs. Colocalization analyses revealed a predominant association of pUS27 or pUL78 with the *trans*-Golgi network or the endoplasmic reticulum, respectively. Intriguingly, our data emphasize that protein sorting is highly regulated by viral functions as we detected dramatic changes in the colocalization of pUS27 and pUL78 with endosomal markers during progression of HCMV replication. Furthermore, we observed cell type-dependent differences in trafficking of both vGPCRs between fibroblasts and epithelial cells. Most importantly, infection experiments with a recombinant HCMV carrying tagged versions of pUS27 and pUL78 simultaneously, revealed that these two proteins do not colocalize during viral infection. This contrasts to results of transient expression experiments. In conclusion, our results highlight the importance to investigate vGPCR trafficking in a viral context.

## 1. Introduction

G protein-coupled receptors (GPCRs) act as key regulators of numerous cellular processes via transmitting the response of various signal molecules like chemokines, hormones, or neurotransmitters. Thus, it is not surprising that viruses have hijacked mammalian GPCRs during coevolution to ensure efficient viral propagation [1,2]. Thereby, virally encoded GPCRs (vGPCRs) illustrate an effective means to bypass the immune system, modulate cellular functions, and redirect cellular signaling networks [3]. Human cytomegalovirus (HCMV) encodes four GPCR homologs, termed pUS27, pUS28, pUL33, and pUL78 [4,5]. In contrast to pUS27 and pUS28, which are restricted to primate CMVs, pUL33, and pUL78 are highly conserved among all betaherpesviruses. While their expression is not crucial for virus replication in cell culture [6,7], a deletion leads to a significantly diminished pathogenesis in animal experiments [8,9].

The most comprehensively analyzed vGPCR of HCMV is pUS28. As a homolog of CCR1, CCR2, and CX3CR1, it can bind a broad spectrum of chemokines, such as CCL5/RANTES, monocyte chemoattractant protein-1 (MCP-1), and CX3CL1/fractalkine [10,11,12]. As pUS28 is rapidly internalized in a constitutive manner [13,14,15,16], it is believed to function as a chemokine sink to impair leukocyte recruitment thereby dampening the immune response at sites of infection [7,17,18,19]. Furthermore, similar to other vGPCRs, pUS28 constitutively activates several signaling pathways including PLCβ [20,21], NF-κB [10], NFAT, and CREB [22] via Gα_q_ and Gα_i_-dependent signaling. Due to the strict host specification of all CMVs, it is difficult to determine the role of pUS28 in a physiologically relevant situation *in vivo*. However, recent reports demonstrated a partial functional complementation between pUS28 and its mouse cytomegalovirus vGPCR homolog pM33 [23,24]. Nevertheless, it is still unknown whether pUS28 plays an essential role during HCMV pathogenesis [7,19].

In contrast to pUS28, the vGPCRs pUS27, pUL78, and pUL33 are still orphan receptors. Interestingly, for pUL33 and its homologs in mouse (pM33) [11] and rat CMV (pR33) [25] a ligand-independent activation of several signaling cascades could be shown [26]. Whereas a functional analysis of pUL33 is difficult *in vivo*, rodent homologs have been reported to be critical for infection [8,27,28]. By means of gene-knockout viruses it was shown that deletion of M33 or R33 resulted in less virulent CMV variants, which neither disseminated nor replicated within the salivary gland. Intriguingly, both pUL33 and pUS28 were able to partially restore the replication defect of a M33-deficient virus indicating shared biological functions between the respective vGPCRs [23].

While pUS28 and pUL33 constitutively activate multiple signaling pathways, the functions of pUS27 and pUL78 are not yet fully understood. As pUS27 and pUS28 are directly adjacent and share 31% sequence identity, a gene duplication event may have occurred. Similar to pUS28, pUS27 is constitutively internalized and localized to cellular vesicles with endosomal markers (EEA1), to lysosomes (LAMP-1), as well as to Golgi compartments (GM130) [29,30]. A potential di-leucine sorting motif in the cytoplasmic domain of pUS27 was reported to be necessary and sufficient for its intracellular localization [29]. The fact that pUS27 is heavily glycosylated [31] and possesses two conserved cysteine residues in the second and third extracellular loop indicates a possible involvement of pUS27 in chemokine binding and signaling in host cells. Notably, O’Connor *et al.* were able to demonstrate that pUS27 supports viral spread through the extracellular route late during infection in fibroblasts and endothelial cells, but not in epithelial cells [32]. Thus, this study suggested a cell type-dependent function of pUS27, which appears to involve interactions with one or more virus-encoded proteins at the site of virus assembly. Moreover, pUL78 was recently shown to support viral infection affecting a step after plasma membrane binding but before virus entry in epithelial cells [33]. This observation was also cell type-specific as pUL78 seemed to be dispensable for virus replication in fibroblasts and endothelial cells. Furthermore, the rodent variants (pR78, pM78) of pUL78 were used to demonstrate the importance of this vGPCR for viral pathogenesis *in vivo* [9,34,35]. In line with the three other vGPCRs of HCMV, pUL78 is constitutively internalized [36]. Wagner *et al.* could show that the process of internalization is dependent on dynamin. In addition, they could identify an association of pUL78 with the endoplasmic reticulum (ER), and its localization in the *trans*-Golgi network as well as early endosomes. Although pUS27 and pUL78 seem to lack constitutive signaling activity [11], they were demonstrated to colocalize and heteromize with the constitutively active receptor pUS28 *in vitro*. Thereby, pUL78, in contrast to pUS27, is able to silence pUS28-mediated activation of NF-κB dependent gene expression [37].

As there is a lack of antibodies against HCMV-encoded GPCRs, most reports assessing the subcellular localization of these proteins were based on transient transfection analyses using tagged receptors. In this study, we describe the construction of recombinant HCMVs expressing either singly tagged pUS27 and pUL78, or a virus, where both proteins were tagged simultaneously, thus, enabling live cell imaging. These viruses were used for a comparative analysis of the subcellular localization of pUS27 and pUL78 after infection of both fibroblasts and epithelial cells. These experiments revealed cell type-dependent differences in trafficking of pUS27 and pUL78. In addition, colocalization analyses with cellular markers showed a substantially different accumulation of both receptors in infected cells compared to transiently transfected cells. Thus, this study emphasizes the necessity to analyze vGPCRs in a viral context in order to obtain physiologically meaningful results concerning their functions during viral replication.

## 2. Results

### 2.1. Colocalization of pUS27 and pUL78 in Transiently Transfected Cells

**Figure 1:**
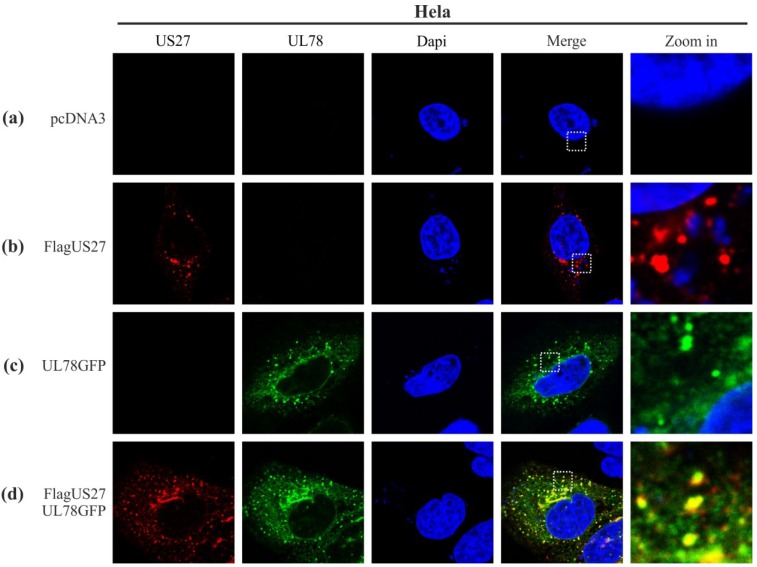
Colocalization analysis of FLAG-US27 and UL78-GFP in transiently transfected HeLa cells. HeLa cells were transfected with 1 μg of an expression plasmid encoding *N*-terminally tagged wild-type TB40/E FLAG-US27 (**b**), *C-*terminally tagged wild-type TB40/E UL78-GFP (**c**), or both plasmids together (**d**). pcDNA3 was used as a negative control (**a**). Cells were fixed 24 h post transfection and permeabilized with Triton X-100. The primary antibody mAb-FLAG in combination with a secondary anti-mouse antibody coupled to Alexa-555 were used to detect pUS27. Cell nuclei were stained with 4',6-diamidino-2-phenylindole (DAPI).

The viral GPCR pUS28 has previously been shown to colocalize and heteromize with pUL78 or pUS27 *in vitro* [37]. In order to investigate a possible colocalization of pUS27 with pUL78 in transiently transfected cells, we used tagged versions of both receptors due to a lack of specific antibodies for pUS27 or pUL78. Thus, a FLAG-tag was fused to the *N*-terminal extracellular domain of pUS27. The *C*-terminal intracellular domain of pUL78 was labeled with GFP. To analyze the intracellular vGPCR localization, receptors were transiently expressed in HeLa cells, either alone or in combination. After 24 h, cells were fixed, permeabilized, stained, and analyzed by confocal microscopy. Both proteins were detected in a predominantly perinuclear distribution pattern. While pUS27 was only found in dot-like structures (Figure 1b, red), pUL78 showed an additional weaker signal distributed throughout the cytoplasm (Figure 1c, green) as already described in previous reports [30,36]. In co-transfection experiments, most of the pUS27 signal overlapped with the dot-like pUL78 signal (Figure 1d). Therefore, both receptors not only colocalize with pUS28 [37], but also show a distinct colocalization among each other presumably in endosomal structures.

### 2.2. Recombinant Viruses Harboring Tagged Versions of pUS27 or pUL78 Show Similar Growth Kinetics Compared to Wildtype HCMV Strain TB40/E

**Figure 2:**
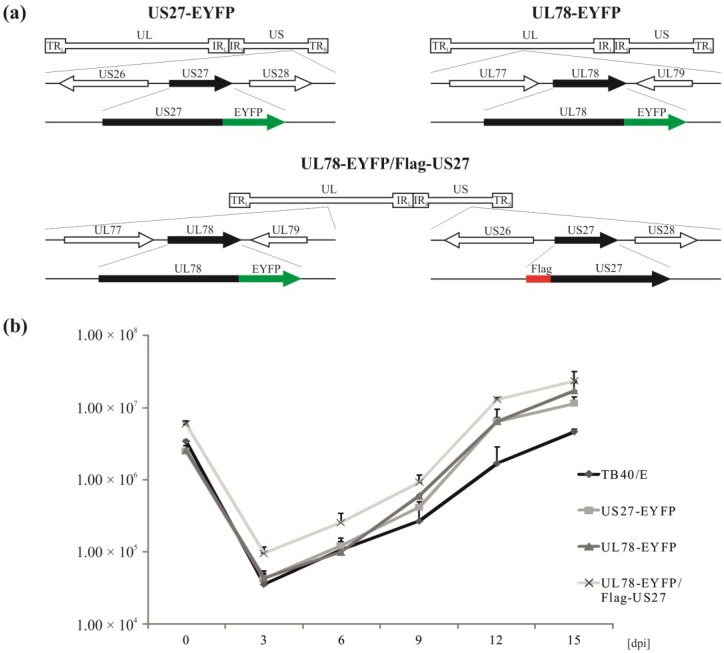
Construction and characterization of recombinant HCMVs expressing tagged US27 and/or UL78. (**a**) Schematic illustration of the US27 and UL78 genomic loci of HCMV strain TB40/E. Recombinant viruses were generated with *C*-terminal EYFP (US27 or UL78, upper panel) or *N*-terminal FLAG-tag (US27, lower panel); (**b**) Growth kinetics of recombinant TB40/E-derived viruses. HFF cells were seeded and infected three days later with wild-type and recombinant viruses at an MOI of 0.1. Virus supernatant samples were harvested at indicated times (dpi, days post infection) and digested with proteinase K. Viral DNA was quantitated via Taqman PCR to determine the release of viral particles from infected cells. Each infection was performed in triplicate, and the standard deviations are shown.

To analyze whether the intracellular localization patterns of pUS27 and pUL78 as observed in transiently transfected cells [29,30,36] can also be detected during infection, recombinant cytomegaloviruses were engineered via homologous recombination. The two-step, red-mediated recombination for markerless DNA manipulation, as described by Tischer *et al.*, was used for the generation of recombinant CMVs [38]. The wild-type TB40/E-based HCMV bacterial artificial chromosome (BAC) TB40-Bac4 [39] was manipulated in order to fuse EYFP to the *C*-terminus of UL78 or US27, resulting in recombinant viruses termed TB40/E-US27-EYFP (US27-EYFP) or TB40/E-UL78-EYFP (UL78-EYFP), respectively (Figure 2a, upper panel). To enable colocalization studies with both vGPCRs in one cell, we additionally generated a recombinant virus carrying tagged versions of US27 and UL78 (Flag and EYFP, respectively), referred to as UL78-EYFP/Flag-US27 (Figure 2a, lower panel). In order to confirm the correct in-frame integration of all tags, BACs were analyzed using various independent methods. Distinct PCR reactions and subsequent nucleotide sequence analyses of all BACs, as well as restriction fragment length polymorphism analysis (RFLP) revealed a correct BAC recombination (data not shown). After reconstitution of infectious viruses, viral growth was analyzed by performing multistep growth curve analyses of wild-type and recombinant TB40/E viruses (Figure 2b). For this purpose, HFFs were infected at an MOI (multiplicity of infection) of 0.1 with either wild-type or recombinant viruses. The supernatants were harvested at 0, 3, 6, 9, 12, and 15 dpi (days post infection). After cell lysis and DNA extraction, real-time PCR was performed to quantify HCMV genome copy numbers. As depicted in Figure 2b, all recombinant viruses replicated with similar growth kinetics compared to the wild-type strain TB40/E. Therefore, the epitope tags do not alter HCMV replication capacities in HFFs. Thus, recombinant viruses can be used to analyze intracellular localization routes of pUS27 and pUL78 during the entire replication cycle, even by live cell imaging.

### 2.3. Expression and Localization Patterns of pUS27 and pUL78 Differ upon HCMV Infection of Fibroblasts and Epithelial Cells

While, up to now, pUS27 localization was only determined in detail in transiently transfected cells [29,30], the intracellular distribution of pUL78 was additionally analyzed at 48 hpi in HFF cells [36]. It has been demonstrated in previous studies that HCMV infection changes the entire morphology of infected cells [40,41]. One of these alterations is the development of a so-called cytoplasmic virion assembly compartment (cVAC). Usually, one cVAC per cell is formed by rearrangement of several cellular compartments including Golgi bodies, the *trans*-Golgi network and endosomal structures [41,42,43]. Thus, the dynamic nature of endocytic compartments during infection compels the use of multiple time points in analyses. Furthermore, it is important to analyze vGPCR localization in different cell types, as HCMV can infect a remarkably broad spectrum of different cell types with variable outcomes for the host *in vivo* [44].

**Figure 3:**
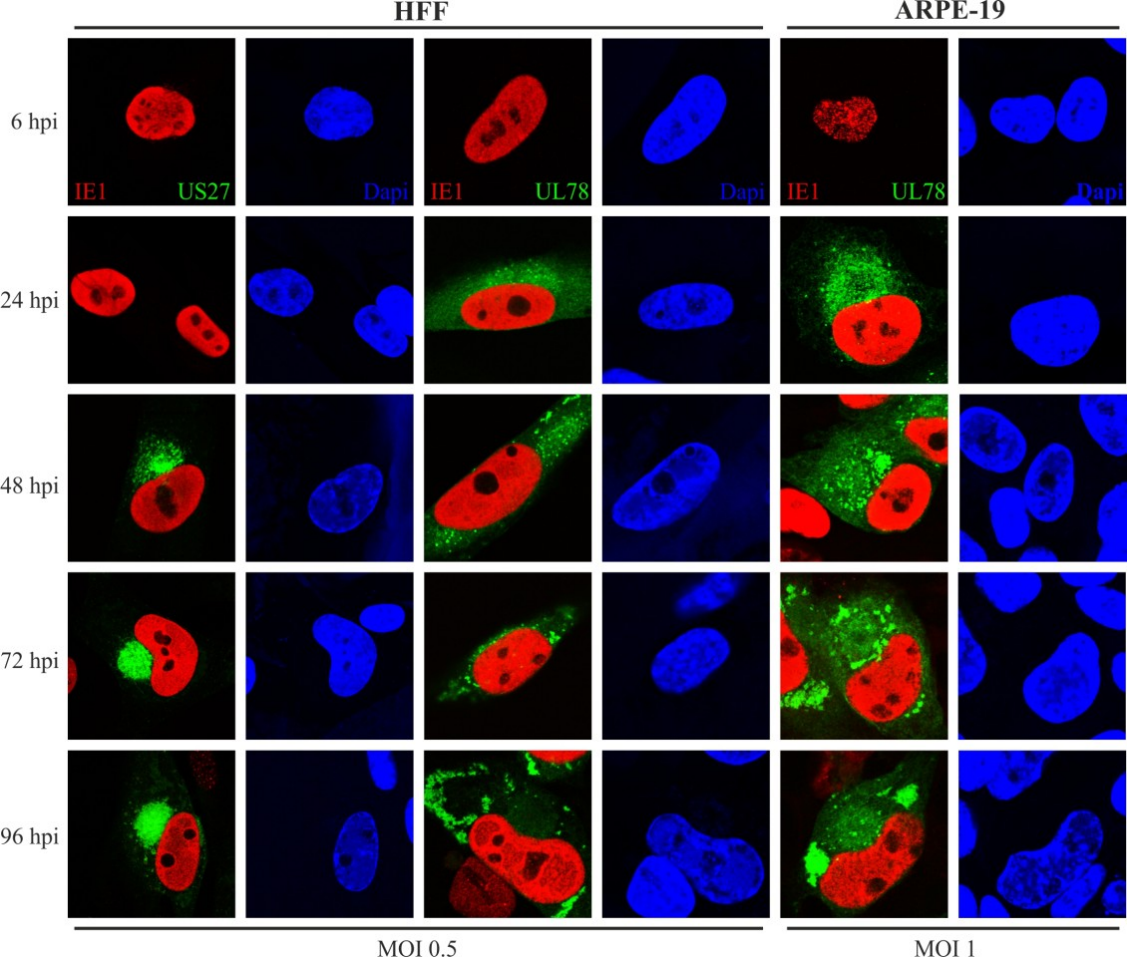
Subcellular localization of US27-EYFP and UL78-EYFP in infected HFFs and ARPE-19 cells. HFF (left and middle panel) or ARPE-19 cells (right panel) were infected with recombinant TB40/E viruses (MOI: 0.5 or 1) expressing fusion proteins of pUS27 (left panel) or pUL78 (middle and right panel) with EYFP and fixed at different time points during HCMV infection (6–96 hpi). Cells were stained with primary antibody mAb-IE1 (p63-27), and a secondary anti-mouse antibody coupled to Alexa-555. Cell nuclei were stained with DAPI.

In a first set of experiments we aimed to determine the subcellular localization of pUS27 and pUL78 in human retinal pigment epithelial cells (ARPE-19) and HFFs over the entire replication cycle. For this, HFFs and ARPE-19 were seeded, infected at an MOI of 0.5 or 1, respectively, and fixed at 6, 24, 48, 72, and 96 h post infection (hpi). After fixation, infected cells were permeabilized, stained for IE1 detection, and analyzed using confocal microscopy. The progression of virus infection in HFF and ARPE-19 cells is shown in Figure 3. To verify that all detected vGPCR signals were specific for infected cells, IE1 staining was used as a control (Figure 3, red). At 6 h after HCMV infection, the IE1 protein was already present in the cell nucleus of HFF and ARPE-19 cells. As described previously [36], pUL78 (Figure 3, green, middle and right panel) was produced between 6 and 24 hpi. In contrast to that, pUS27 (Figure 3, green, left panel) expression was firstly visible 48 h after infection. Thus, it appears to be expressed later during HCMV replication. During the entire replication cycle, pUS27 was present in the perinuclear region of the cell. Late in infection, however, pUS27 was not only detected in association with the cVAC, but was additionally found in dot-like structures all over the cytoplasm, which was both observed in HFF and ARPE-19 cells (data not shown). In contrast to the perinuclear distribution of pUS27 at 48 hpi, the pUL78 signal was spread over the entire cytoplasm including defined dot-like structures. As infection progressed (72–96 hpi), pUL78 was increasingly displaced from the cVAC formation site, both in HFF and in ARPE-19 cells. Nevertheless, in epithelial cells dot-like pUL78-positive structures remained in the perinuclear region over the entire replication cycle. This observation suggests that pUL78 may exhibit different functions during infection of epithelial cells versus fibroblasts.

### 2.4. While Both Receptors Localize to the *trans*-Golgi Network in Virus Infected Cells, only pUL78 Colocalizes with Calreticulin

**Figure 4:**
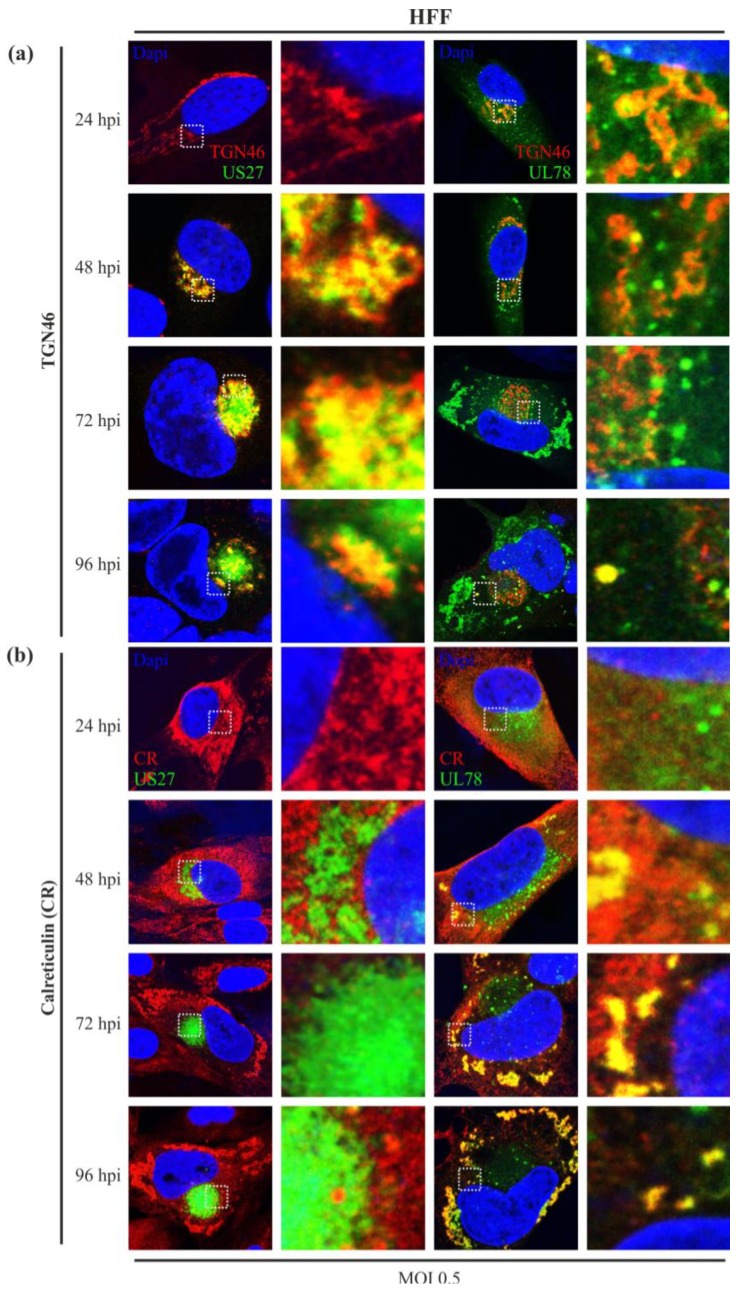
Colocalization of US27-EYFP and UL78-EYFP with TGN46 and calreticulin in infected HFFs. HFFs were infected with recombinant TB40/E viruses (MOI: 0.5) expressing fusion proteins of pUS27 (left panel) or pUL78 (right panel) with EYFP and fixed at different time points during HCMV infection (24–96 hpi). Cells were stained with primary antibody (**a**) pAb-TGN46, diluted 1:300, or (**b**) pAb-Calreticulin (PA3-900), diluted 1:100, and the respective secondary anti-sheep antibody or anti-rabbit antibody coupled to Alexa-555 (1:400). Cell nuclei were stained with DAPI.

To better define the intracellular localization patterns of pUS27 and pUL78 during HCMV infection, we performed indirect immunofluorescence analysis using different cellular markers. In a first experiment, HFF and ARPE-19 cells were infected with either US27-EYFP or UL78-EYFP to identify a potential colocalization with proteins of the Golgi network or the endoplasmic reticulum. Since many vGPCRs, including pUS27, are predicted to be highly glycosylated [31], we expected a colocalization with the *cis*-Golgi marker GM130 in HFF as well as in ARPE-19 cells. In contrast to previous reports [29,45], we were not able to visualize any colocalization with GM130 in infected cells over the entire replication cycle (data not shown). However, in accordance with transiently transfected receptors [30,36], partial colocalization of pUS27 and pUL78 was detected with the membrane protein TGN46, a marker for the *trans*-Golgi network, during the progression of HCMV infection (Figure 4a). Similar to GM130, the TGN46 signal showed perinuclear localization early in infection (Figure 4a, 24 hpi). After 48 h, the signal shifted to the cVAC formation site and started forming concentric circles around this virus-induced, cellular structure (Figure 4a, 72 hpi). Upon pUS27 expression, the vGPCR localizes to the *trans*-Golgi network (TGN) (Figure 4a, left panel), suggesting that the TGN serves as site of glycosylation. This expression pattern lasted until late stages of HCMV infection. Upon comparison of these findings to pUL78, we observed a slightly different pattern. As pUL78 was detected throughout the cytoplasm at early stages (Figure 4a, 6 hpi, right panel) and was almost excluded from the virion assembly site late in infection (Figure 4a, 72 hpi, right panel), we detected a partial colocalization of TGN46 with pUL78 but not as striking as with pUS27. Next, we investigated a potential colocalization of both vGPCRs during infection with calreticulin, a marker for the endoplasmic reticulum (ER) (Figure 4b). At early stages of virus infection, calreticulin showed a typical lamellae structure formed around the cell nucleus. After approximately 48 to 72 h, the structure changed and was completely displaced from the site of cVAC formation. As pUS27 is associated with the cVAC, we did not observe any colocalization of this receptor with calreticulin. In contrast to pUS27 (Figure 4b, left panel), pUL78 appeared to be associated with the ER over the entire replication cycle of HCMV (Figure 4b, right panel). The same results were obtained in ARPE-19 cells (data not shown). These results are in accordance with previous studies using transiently transfected cells [36].

### 2.5. Endosomal Localization Patterns of pUS27 and pUL78 Diverge in Fibroblasts as Well as in Epithelial Cells during HCMV Replication

**Figure 5:**
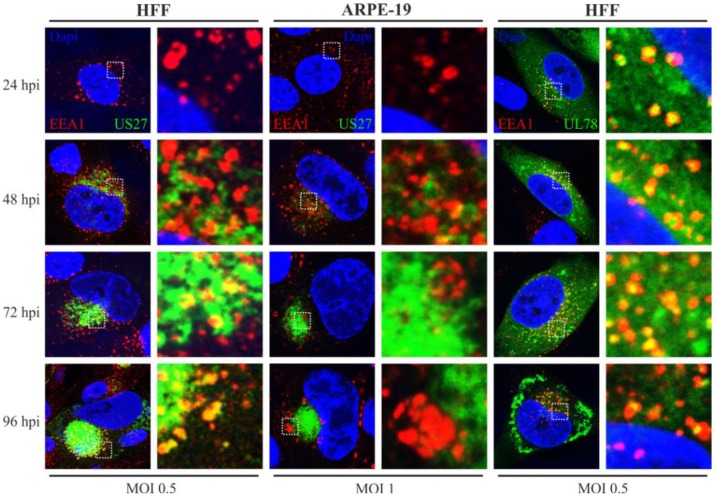
EEA1 staining of US27-EYFP and UL78-EYFP infected HFFs and ARPE-19 cells. HFF (left and right panel) or ARPE-19 cells (middle panel) were infected with recombinant TB40/E viruses (MOI: 0.5 or 1) expressing fusion proteins of pUS27 (left and middle panel) or pUL78 (right panel) with EYFP and fixed at different time points during HCMV infection (24–96 hpi). Cells were stained with primary antibody pAb-EEA1 (H-300), diluted 1:200, and the secondary anti-rabbit antibody coupled to Alexa-555 (1:400). Cell nuclei were stained with DAPI.

**Figure 6:**
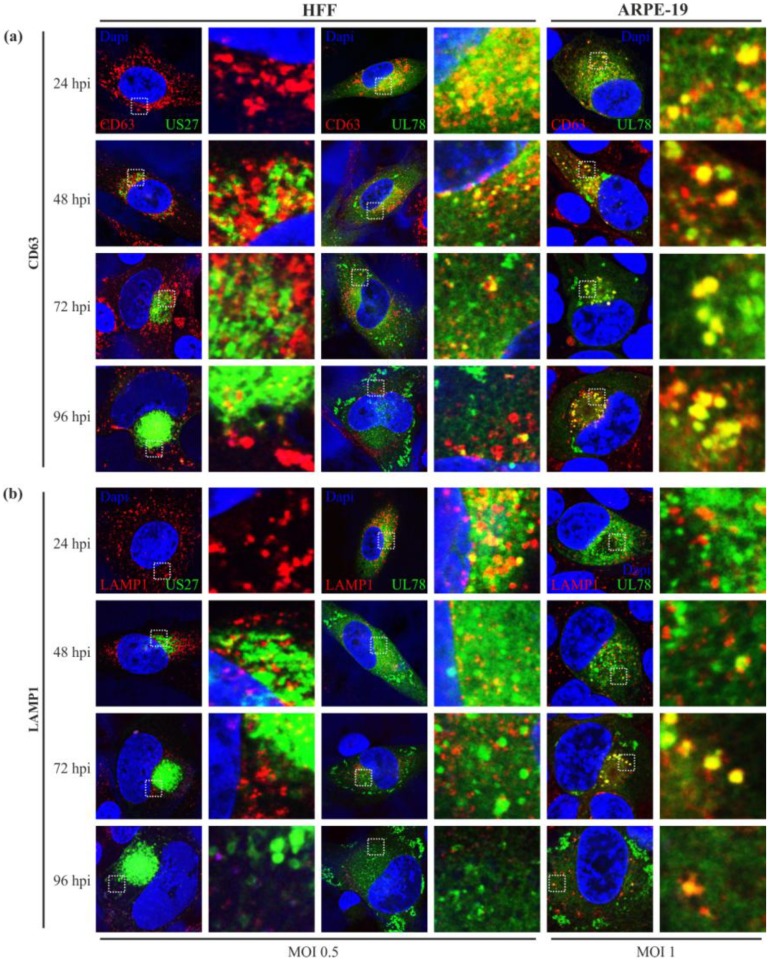
CD63 and LAMP1 staining of US27-EYFP and UL78-EYFP infected HFFs and ARPE-19. HFF (left and middle panel) or ARPE-19 cells (right panel) were infected with recombinant TB40/E viruses (MOI: 0.5 or 1) expressing fusion proteins of pUS27 (left panel) or pUL78 (middle and right panel) with EYFP and fixed at different time points during HCMV infection (24–96 hpi). Cells were stained with primary antibody (**a**) mAb-CD63 (MX-49.129.5) or (**b**) mAb-LAMP-1 (H5G11), each diluted 1:50, and the secondary anti-mouse antibody coupled to Alexa-555 (1:400). Cell nuclei were stained with DAPI.

Next, we determined whether there is any colocalization of pUS27 or pUL78 with endosomal markers during the time course of HCMV infection. In order to describe endosomal structures in detail, colocalization studies with different endosomal and lysosomal markers were performed. The main components of the endosomal system are early endosomes (EE), recycling endosomes (RE), maturing endosomes (ME), late endosomes (LE), and lysosomes. During cVAC formation, components of the host cell secretory apparatus are rearranged in concentric circles [41]. Thereby, early (EEA1) and recycling (CD71) endosomes were localized in the cVAC center and were enclosed with markers of the *trans*-Golgi and *cis*-Golgi network in HFFs (Figure 4 and Figure 5). Markers of the late endocytic pathway (CD63, LAMP-1), however, were found in the cVAC periphery (Figure 6). For pUL78, a partial colocalization with EEA1 was already shown at 48 h post infection in a previous report [36]. Here, we demonstrate that co-localization of pUL78 signal with that of EEA1 is not restricted to the 48 h post infection time point, but in fact occurs throughout the HCMV replication cycle in HFFs (Figure 5, right panel) as well as in ARPE-19 cells (data not shown). In contrast, the pUS27 signal colocalized with EEA1 in HFFs only at late times of infection (Figure 5, left panel). Intriguingly, no colocalization between pUS27 and EEA1 could be detected in ARPE-19 cells, infected with the same recombinant virus (Figure 5, middle panel). Thus, not only pUL78 but also pUS27 may exhibit differential functions in HFFs versus ARPE-19 cells.

The endosomal machinery is a highly complex system. After internalization and bypass of early endosomes, cargo can enter a recycling circuit or a degradative system, in which endosomes mature to MEs, and later to LEs [46]. To further elucidate the fate of HCMV-encoded vGPCRs after internalization, we used markers for REs (CD71), MEs/LEs (CD63), and lysosomes (LAMP1). We expected a colocalization with recycling endosomes, as both receptors were shown to localize to the cell surface [29,30,36]. Unfortunately, no overlap of vGPCR signals with CD71 could be detected in any cell type (data not shown). This may suggest that pUS27 and pUL78 recycling is independent of CD71-positive endosomes occurring directly via EEs. Alternatively, both receptors might enter the cellular degradative system immediately after internalization. To address this hypothesis, we next focused on maturing endosomes, late endosomes and lysosomes. CD63, a marker for multivesicular bodies, appeared as dot-like structures (Figure 6a), which were widely spread over the cytoplasm early in HCMV infection (24 hpi). After 48 h, the signal began to concentrate in the perinuclear region. Interestingly, CD63 signal seemed to be downregulated during cVAC formation in HFFs (Figure 6a, left and middle panel), as described previously [43]. In ARPE-19 cells, however, we could not observe any changes in CD63 expression (Figure 6a, right panel). Similar to EEA1, pUL78 began to colocalize with CD63 at 24 hpi (Figure 6a, middle and left panel). This colocalization could be observed throughout the replication cycle in HFFs and ARPE-19 cells and was decreased with the reduction of the CD63 signal in HFFs late in infection (Figure 6a, middle panel). In contrast, pUS27 did not display any colocalization with the cellular marker CD63 (Figure 6a, left panel), indicating that this receptor is not present in mature and late endosomes of infected HFFs and ARPE-19 cells (data not shown). Corresponding to CD63, LAMP1, a marker for lysosomes, could be recognized in dot-like structures widely spread throughout the cytoplasm at 24 hpi; at 48 hpi, the signal started to concentrate in the perinuclear region of the cell (Figure 6b). Furthermore, pUS27 was not observed in lysosomes in HFFs (Figure 6b, left panel) or ARPE-19 cells (data not shown). Nevertheless, pUL78 signal overlapped with LAMP1 (Figure 6b, middle and right panel). This pattern, however, differed strongly dependent on the cell type. While we could demonstrate that pUL78 was localized to lysosomes early in infection in HFFs (Figure 6b, middle panel), the dot-like signals of pUL78, located close to the nucleus, showed clear colocalization with LAMP-1 in ARPE-19 cells starting at 72 h post infection (Figure 6b, right panel).

### 2.6. pUS27 and pUL78 Do Not Colocalize in Infected Fibroblasts or Epithelial Cells throughout the Replication Cycle

**Figure 7:**
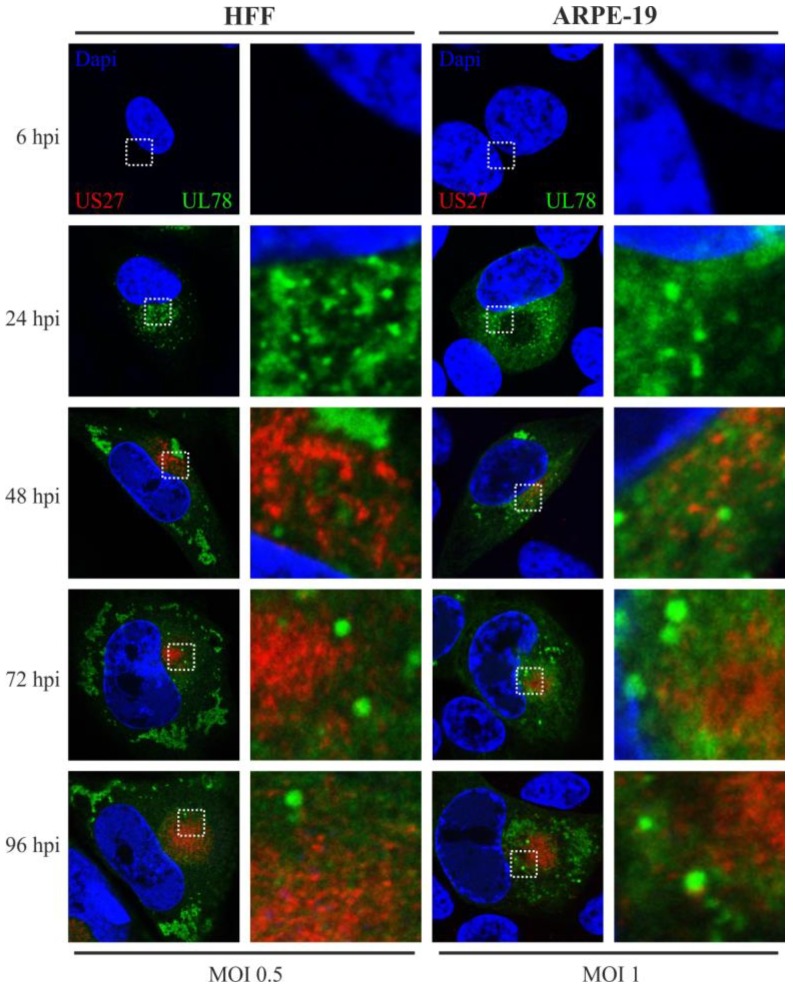
Colocalization analysis of Flag-US27 and UL78-EYFP in infected HFFs and ARPE-19 cells. HFF (left panel) or ARPE-19 cells (right panel) were infected with a recombinant TB40/E virus (MOI: 0.5 or 1) expressing fusion proteins of both pUS27 (red) and pUL78 (green) with Flag or EYFP, respectively, and fixed at different time points during HCMV infection (24–96 hpi). Cells were stained with primary antibody mAb-Flag, diluted 1:400, in combination with the secondary anti-mouse antibody coupled to Alexa-555 (1:400) in order to detect pUS27. Cell nuclei were stained with DAPI.

Finally, we set out to clarify whether there is any colocalization of both vGPCRs in one cell during HCMV infection. To that end, a recombinant virus carrying tagged versions of the US27, as well as the UL78 gene (Flag/EYFP), was engineered by means of BAC technology (Figure 2) and investigated by immunofluorescence analysis. Colocalization studies with variable cellular markers already revealed that localization patterns of pUS27 and pUL78 differ substantially. Here, we demonstrate for the first time that pUS27 and pUL78 do not display any colocalization throughout the HCMV replicative cycle. This observation was independent of the cell type, as both vGPCR signals did not overlap in HFFs or ARPE-19 cells (Figure 7). In conclusion, this strongly suggests that the localization patterns of virally-encoded GPCRs as determined in transiently transfected cells do not reflect the physiological patterns during infection. Our data highlight the importance to analyze vGPCR functions in a viral context.

## 3. Discussion

Human cytomegalovirus (HCMV), as all herpesviruses, establishes a lifelong infection in the host. In order to maintain its persistent state, HCMV developed various mechanisms during coevolution with its host to modify the cellular signaling network and to evade the immune system. It is likely that the expression of four homologs of cellular seven-transmembrane receptors termed pUS27, pUS28, pUL33, and pUL78 contributes to the dysregulation of cellular signaling and to immune evasion [4,5]. While cellular G-protein coupled receptors (GPCRs) typically localize to the cell surface and induce intracellular signaling cascades upon ligand binding [47], several reports of viral GPCRs (vGPCR) showed a different receptor localization. For instance, in the absence of ligands pUS28 was predominantly found in an intracellular pattern in endosomal compartments [15]. Similar observations were made for the orphan receptors pUS27 and pUL33, where the majority of the protein was detected in the perinuclear region of cells [30]. Moreover, the vGPCR pUL78 seemed to associate mainly with the endoplasmic reticulum (ER) [36]. Unfortunately, most of these results were obtained with transiently expressed receptors. To gain more insight into the subcellular localization of HCMV-encoded vGPCRs during viral infection we performed immunofluorescence analyses of recombinant HCMVs, based on the endotheliotropic strain TB40/E, expressing either EYFP- or Flag-tagged pUS27 and/or pUL78.

In accordance with previous studies, we could confirm that pUL78 has an early/late expression pattern [48], whereas pUS27 is expressed later during infection. While pUS27 exhibits an intracellular perinuclear distribution and is recruited to the cytoplasmic virion assembly complex (cVAC), the early protein pUL78 is observed throughout the cytoplasm. Due to stable colocalization with the ER, which is known to be excluded from the cVAC formation site [41,42], pUL78 is completely displaced from the virus assembly zone in HFFs. Importantly, our results indicate pronounced variations in localization patterns of virally-encoded GPCRs compared to transiently expressed pUS27 or pUL78. Contrary to transiently expressed vGPCRs [29,45], we were not able to visualize any colocalization with the *cis*-Golgi marker GM130 in HFFs or ARPE-19 cells (data not shown). Similar to recent reports [30,36,45], a colocalization of pUS27 and pUL78, with TGN46, a marker for the *trans*-Golgi network (TGN), was obvious and stable over the whole replication cycle. Thus, in contrast to Stapleton *et al.* [29], we suggest the TGN as the site of receptor glycosylation for at least pUS27.

Furthermore, we detected a clear colocalization of pUL78 with EEA1 immediately upon expression, whereas pUS27 started to colocalize with the marker for early endosomes (EEs) at late time points after infection (96 hpi) only in HFFs. This observation differed significantly from the pattern determined for transiently expressed receptors [29,36]. Our data provide evidence that protein sorting is a dynamic process leading to dramatic changes of receptor localization during the entire HCMV replication cycle. Thus, it is of obvious importance to investigate protein localization patterns in a viral context. A surprising finding was that neither pUS27 nor pUL78 signal overlapped with CD71, a marker for recycling endosomes (REs). Both receptors were reported to constitutively internalize [29,30,36]. Hence, we expected a colocalization with REs, which was previously shown for the pUL78 homolog pM78 [45]. However, this result does not necessarily exclude pUS27 and pUL78 recycling in infected cells, since receptor recycling may also occur via a direct route from EEs, the so called fast recycling pathway [49], or, as shown for cellular GPCRs, via the TGN [50]. On the other hand, both vGPCRs could enter a cellular degradative system directly after internalization [46]. In contrast to transiently expressed pUS27 [30], we were not able to visualize any colocalization with the cellular markers CD63 and LAMP1. Only pUL78 extensively overlapped with these late endosomal (LE) and lysosomal markers, contrary to a transiently transfected version [36]. Since Das *et al.* suggested that lysosomes do no longer act as degradation system in infected cells [42], we hypothesized that pUL78 is not degraded but may fulfill a so far unknown function within these structures. However, the colocalization pattern of pUL78 with LEs and lysosomes varied extensively when analyzing different cell types or time points post infection. Consequently, these data again emphasize the highly dynamic protein localization pattern during HCMV replication.

Intriguingly, the most pronounced divergence was detected with our recombinant virus carrying tagged versions of pUS27 and pUL78 simultaneously. In general, it is important to keep in mind that heteromerization of vGPCRs can alter their functions [51]. For example, Tschische *et al.* showed that heterodimerization of pUL78 with pUS28 led to an almost complete block of pUS28-mediated constitutive NF-κB activation [37]. Besides, pUL78 as well as pUL33 were shown to heteromize with the human CCR5 and CXCR4 receptors thereby modifying GPCR functions and cell surface localization [52]. Thus, we hypothesized that a closer investigation of pUS27/pUL78 heterodimerization might unravel novel roles during virus infection and pathogenesis. Unexpectedly, however, we were not able to visualize any overlap of pUS27 and pUL78 signals in infected cells whereas this was clearly detectable in transient transfection experiments (compare Figure 1 and Figure 7). Consequently, recent heteromerization studies of HCMV-encoded vGPCRs have to be interpreted with caution since the subcellular localization of virally-encoded GPCRs as determined in transiently transfected cells may not reflect the physiological patterns during infection.

In accordance with previous studies [32,33], comparative analyses of fibroblasts and epithelial cells revealed a cell type-dependent trafficking of both pUS27 and pUL78. Moreover, we demonstrate that the subcellular morphology changes not only during infection, but also varies between infected HFFs and ARPE-19 cells. Whereas, as described previously [43], CD63 was downregulated during cVAC formation in HFFs, we did not observe any changes in CD63 expression in ARPE-19 cells. This observation was in agreement with a HFF-specific, decreased colocalization of CD63 with pUL78 late during infection. Similar results were obtained for LAMP1. In addition, not only the colocalization of pUL78 varied between cell types, but also its cytoplasmic distribution: at the beginning of HCMV replication, pUL78 signal was spread throughout the cytoplasm in both analyzed cell types; however, a displacement from the cVAC formation site was only observed in HFFs. In ARPE-19 cells additional dot-like pUL78-positive structures remained in the perinuclear region for the entire replication cycle.

Surprisingly, no colocalization of pUS27 with any endosomal marker was detected in ARPE-19 cells. This raises the question whether pUS27 is localized at the cytoplasmic membrane in epithelial cells. A possible explanation would be that pUS27 internalization occurs via EEA1-negative endosomes, for example APPL-positive ones [53], or in a retrograde manner via the TGN [50]. Nonetheless, further investigations will be required to characterize the sorting abilities of pUS27 in more detail.

In conclusion, the results of this study emphasize the importance of analyzing vGPCR sorting in a viral context. Furthermore, we observed an unexpected, cell type-dependent variation in vGPCR receptor localization and sorting suggesting major functional differences of these molecules in terms of their signaling capabilities in various cell types.

## 4. Experimental Section

### 4.1. Cells

HeLa cells were cultivated in Eagle’s minimal essential medium (MEM) (GIBCO/BRL) containing 7% fetal calf serum at 37 °C and 5% CO_2_. Primary human foreskin fibroblasts (HFFs) and the human retinal pigment epithelial cell line ARPE-19 were maintained in MEM, supplemented with 10% fetal calf serum at 37 °C and 5% CO_2_.

### 4.2. Construction of Vectors for Subcellular Localization Analyses

For the FLAG-US27 construct, a FLAG-epitope was fused to the 5' region of the US27 gene (based on TB40/E) after amplification of the US27 coding sequence with primers N-US27-BamHI-for (5'-CTA GGG ATC CAC CAC CTC TAC AAA CCA AAC CTT AAC-3') and N-US27-XhoI-rev (5'-CTA GCT CGA GTT ACA ATA GAA ATT CCT CCT CCC CG-3') followed by insertion into the pcDNA3-derived vector pHM971. For the UL78GFP construct, the GFP coding sequence was fused to the 3' region of the UL78 gene (based on TB40/E): after amplification of the UL78 coding sequence with primers UL78-EcoRI-for (5'-CTG AAT TCA TGT CCC CTT CTG TGG AGG AGA C-3') and 2.UL78-BamHI-2nt-rev (5'-TCG GAT CCT GTA ATG CCG TCA CCG TTG CGT CG-3'), the amplicon was inserted into pEGFP-C1 (Clontech, Heidelberg, Germany), resulting in the expression of a *C-*terminally GFP-tagged pUL78.

### 4.3. Transient Expression Analyses and Protein Localization

For immunofluorescence analyses, 4 × 10^5^ HeLa cells/well were seeded in MEM medium without antibiotics on coverslips until they were grown to approximately 80% confluence. Transfection of DNA plasmids (2 μg total, with or without pcDNA3/well) was performed by utilizing LipofectamineTM 2000 (Invitrogen, Karlsruhe, Germany) according to the manufacturer’s instructions. At 24 h post-transfection, cells were washed three times with PBSo and fixed by incubation with 4% PFA for 10 min at room temperature. After fixation, cells were washed again three times with PBSo and permeabilized with 0.2% Triton X-100 in PBSo at 4 °C for 15 min. After three additional washing steps with PBSo, the MAb-Flag (M2 F1804, Sigma-Aldrich, Deisenhofen, Germany), to stain FLAG-US27, diluted 1:400 in PBSo, was applied and cells were incubated for 1 h at 37 °C. Excessive antibodies were removed by washing three times with PBSo, followed by incubation with anti-mouse Alexa555-conjugated secondary antibody, which was diluted 1:400 in PBSo, and incubated with the cells for a further 45 min at 37 °C. Cells were mounted using the DAPI Hard-set (4,6-Diamino-2-phenylindol)-containing Vectashield mounting medium (Vector Laboratories, Burlingame, CA, USA) and then analyzed using the Leica TCS SP5 confocal laser scanning microscope (Leica, Wetzlar, Germany). Adobe Photoshop package [54] was used for processing of images.

### 4.4. Viruses, Generation of Recombinant HCMVs, and Growth Curve Analysis

All HCMV strains were reconstituted and propagated on human foreskin fibroblasts (HFF) as described previously [38,55] and were based on the HCMV bacterial artificial chromosome (BAC) TB40-Bac4, which was derived from the HCMV strain TB40/E, isolated on endothelial cells [39]. For *C*-terminal and/or *N*-terminal fusion of EYFP- or FLAG-tag to the coding region of US27 and/or UL78 (for details see Figure 2a) a markerless BAC mutagenesis according to Tischer *et al.* [55] was performed in the *E. coli* strain GS1783 (kindly provided by Tischer, B.K., Free University of Berlin, Berlin, Germany). For that purpose, linear recombination fragments were generated by amplification of an I-SceI-aphAI cassette from plasmid pEP-S/aphAI (for FLAG-fusion, kindly provided by Tischer, B.K., Free University Berlin, Berlin, Germany) or plasmid pHM3366 (based on pEP-S/aphAI, for EYFP-fusion) via Hot Start PCR with the polymerase Phusion Hot Start (Thermo Fisher Scientific, Waltham, MA, USA) and specific primer pairs (Table 1). For *N*-terminal fusion of the Flag-epitope, two consecutive PCRs had to be performed. Afterwards, the kanamycin cassette was removed by induction of I-SceI followed by a second Red recombination. Resulting BAC clones, termed TB40/E-US27-EYFP (US27-EYFP), TB40/E-Flag-US27 (Flag-US27), or TB40/E-UL78-EYFP (UL78-EYFP), were verified by distinct PCR reactions and subsequent nucleotide sequence analyses, as well as restriction fragment length polymorphism analysis (RFLP, using restriction endonuclease EcoRV-HF, NEB). To generate double-tagged TB40/E-UL78-EYFP/Flag-US27 (UL78-EYFP/Flag-US27), the parental BAC TB40/E-Flag-US27 (Flag-US27) was used to transform *E.coli* GS1783. Recombination occurred as above with a linear recombination fragment of UL78-EYFP. For virus reconstitution, BAC DNA was isolated from bacteria using the BAC preparation protocol of the PureLink HiPure Plasmid Maxiprep kit (Invitrogen, Karlsruhe, Germany) and transfected into HFFs using X-tremeGENE transfection reagent (Roche, Mannheim, Germany) according to the manufacturer’s instructions. Transfected cells were incubated at 37 °C until plaques appeared, while medium was changed once a week. The supernatant was used for infection of fresh HFF cultures and preparation of virus stocks.

In order to determine the replication capacities of all reconstituted recombinant viruses, multistep growth curve analyses were performed. Therefore, 3 days after seeding, 3.0 × 10^5^ resting HFFs were infected with equal IE1 units (MOI = 0.1) of wild-type TB40/E or recombinant viruses. The viral supernatants were harvested at 0, 3, 6, 9, 12, and 15 days post infection (Figure 2b, dpi, days post infection). Aliquots of the supernatants were treated with proteinase K. Afterwards, to quantify the HCMV genome copy numbers, all samples were subjected to quantitative Real-Time PCR using fluorescence-labeled Taqman probes and the ABI Prism 7500 sequence detector (Applied Biosystems, Foster City, CA, USA) as well as the corresponding software SDS [56]. The viral load was determined by amplification of a region within the exon 4 of the IE1 gene locus (ORF UL123) using the primers CMV5' (5'-AAG CGG CCT CTG ATA ACC AAG-3') and CMV3' (5'-GAG CAG ACT CTC TCA GAG GAT CGG-3') together with the fluorescence labeled probe CMV MIE FAM /TAMRA (5'-CAT GCA GAT CTC CTC AAT GCG GCG-3'). Each infection was performed in triplicate.

**Table 1:** Oligonucleotide primers for generation of recombinant human cytomegalovirus (HCMV) strains.

Name	Construct	Sequence in 5'-3'
Flag-kTischer-fw	Flag-US27 PCR1	GAT TAC AAG GAT GAC GAC GAT AAG
Flag-US27-rev		CAT ATC aga att ggt taa ttg gtt ggt aac act acc tgt aag gtg atg gat tac aag gat gac gac gat aag atg acc acc tct aca aac caa acc tta aca cag gtg agc aac atg aca aa
Flag-kUS27-rev	Flag-US27 PCR2	ttt gtc atg ttg ctc acc tgt gtt
Flag-US27-fw		tgt tat gct ttt tac agg acc gtt cag cag gta aca cta cct gta agg tga tgg att aca agg atg acg acg ata ag
US27-EYFP-fw	US27-EYFP	atg aca gaa aaa atg cac cta tgg agt ccg ggg agg agg aat ttc tat tga tgg tga gca agg gcg agg agc tg
US27-EYFP-rev		gtg caa tga gca aaa ata gat gtg cgg cgg acg cgt gaa aga gga tcg aat tac ttg tac agc tcg tcc atg ccg ag
UL78-EYFP-fw	UL78-EYFP	gca ccg acg gcg aaa aca ccg tcg cgt ccg acg caa cgg tga cgg cat taa tgg tga gca agg gcg agg agc tg
UL78-EYFP-rev		gac gtg att tat ctg cca ctt ttc tcc ccg ctg ccg tac agc gcc gcc gct tac ttg tac agc tcg tcc atg ccg ag

### 4.5. HCMV Infection and Protein Localization

HFF (2 × 10^5^ cells/well) or ARPE-19 cells (4.5 × 10^5^ cells/well) were seeded on coverslips for infection with recombinant viruses. Indirect immunofluorescence analysis was performed as described before for transiently transfected cells. However, after permeabilization, an additional blocking step was performed. Therefore, cells were blocked with human or goat serum diluted 1:10 in PBSo for polyclonal or monoclonal antibodies, respectively, and incubated for 15 min at 37 °C. Afterwards, cells were stained with specific primary antibodies (Table 2) for 1 h at 37 °C and appropriate secondary antibodies Alexa-555 anti-sheep, Alexa-555 F(ab)'2 anti-mouse, or Alexa-555 F(ab)'2 anti-rabbit (Invitrogen, Karlsruhe, Germany) for 45 min at 37 °C and mounted using the DAPI Hard-set-containing Vectashield mounting medium (Vector Laboratories, Burlingame, CA, USA). Images were acquired as before, using a Leica TCS SP5 confocal laser scanning microscope and finally analyzed using Adobe Photoshop package [54].

**Table 2:** Antibodies used in this study.

Target protein	Localization	Dilution	Nature of antibody (source)
IE1	nucleus	1:4	MAb, clone p63–27 [57]
TGN46	*trans*-Golgi network	1:300	Sheep polyclonal antibody (AbD Serotec catalog No. AHP500G)
calreticulin	endoplasmic reticulum	1:100	Rabbit polyclonal antibody (Thermo Fisher catalog No. PA3-900)
EEA1	early endosomes	1:200	Rabbit polyclonal antibody, clone H-300 (SCBT catalog No. 33585)
CD63	multivesicular bodies, late endosomes	1:50	MAb, clone 4X-49.129.5 (SCBT catalog No. 5275)
LAMP1	lysosomes	1:50	MAb, clone H5G11 (SCBT catalog No. 18821)

## 5. Conclusions

In conclusion, our data demonstrate that trafficking of the HCMV-encoded GPCRs pUS27 and pUL78 occurs in a cell type-dependent manner suggesting that both receptors may exhibit functional differences in various cell types. Furthermore, we could clearly show that pUS27 as well as pUL78 trafficking are affected by additional viral proteins. Thus, analysis of pUS27 and pUL78 in a viral context is mandatory in order to further decipher the functions of these two vGPCRs during HCMV replication.
